# Cognitive training for children and adolescents with fragile X syndrome: a randomized controlled trial of Cogmed

**DOI:** 10.1186/s11689-019-9264-2

**Published:** 2019-04-15

**Authors:** David Hessl, Julie B. Schweitzer, Danh V. Nguyen, Yingratana A. McLennan, Cindy Johnston, Ryan Shickman, Yanjun Chen

**Affiliations:** 1 MIND Institute, University of California Davis Medical Center, 2825 50th St, Sacramento, CA 95817 USA; 2 Department of Psychiatry and Behavioral Sciences, University of California Davis School of Medicine, 2230 Stockton Blvd, Sacramento, CA 95817 USA; 3 Department of Pediatrics, University of California Davis School of Medicine, 2516 Stockton Blvd, Sacramento, CA 95817 USA; 4 Department of Medicine, University of California Irvine, 333 City Blvd. West, Orange, CA 92868 USA; 5 Institute for Clinical and Translational Science, Irvine, CA 92697 USA; 6 Translational Psychophysiology and Assessment Laboratory (T-PAL), MIND Institute, UC Davis Medical Center, 2825 50th Street, Sacramento, CA 95817 USA

**Keywords:** Fragile X mental retardation protein, *FMR1* gene, Intellectual disability, Treatment, Working memory

## Abstract

**Background:**

Individuals with fragile X syndrome (FXS) typically demonstrate profound executive function (EF) deficits that interfere with learning, socialization, and emotion regulation. We completed the first large, non-pharmacological controlled trial for FXS, designed to evaluate the efficacy of Cogmed, a computer/tablet-based working memory (WM) training program.

**Methods:**

The study was a randomized, blinded, parallel two-arm controlled trial in 100 children and adolescents with FXS (63 male, 37 female; 15.28 ± 3.36 yrs.). Participants were randomized equally to adaptive (difficulty level adjusted to performance) or non-adaptive (control) Cogmed training. Participants were assessed at home using objective measures of WM (primary outcome) and EF at baseline, following 20–25 caregiver-supported sessions over 5–6 weeks, and at follow-up 3 months after cessation of training. Parents and teachers provided ratings of WM, attention, and EF.

**Results:**

The WM composite and selective domains of EF (distractibility, cognitive flexibility), as well as parent- and teacher-reported attention and EF, significantly improved across the full study sample, with many changes maintained at follow-up. However, comparisons of improvement between adaptive and non-adaptive control conditions did not differ, showing that progressively challenging the WM system by expanding span length did not provide added benefit overall.

**Conclusions:**

Further experimental comparisons are needed before Cogmed working memory training can be considered empirically validated for children with FXS, forming the basis of treatment recommendation. However, given that prior studies show no significant changes on these measures in FXS without treatment, that improvements were maintained for 3 months, and that blinded teachers reported improvements in the classroom, the modest benefits seen in both adaptive and non-adaptive groups overall are unlikely to be attributable to placebo or practice effects alone. Future analyses examining inter-individual differences (e.g., baseline capacity, training efficiency, co-morbidity, training environment, characteristics of training aide) may help to link this intervention to outcomes and potential transfer effects.

**Trial registration:**

US National Institutes of Health (ClinicalTrials.gov), NCT02747394.

**Electronic supplementary material:**

The online version of this article (10.1186/s11689-019-9264-2) contains supplementary material, which is available to authorized users.

## Background

Fragile X syndrome (FXS) is caused by a so-called “full mutation” in the fragile X mental retardation 1 (*FMR1*) gene at Xq27.3 and occurs in an estimated 1 of every 2500 to 5000 live births [1]. It is the most common inherited cause of intellectual disability, and over 90% of males and 30–50% of females with the full mutation have IQ scores in this range (IQ < 70) [2]. The cognitive phenotype of FXS is characterized by prominent deficits in executive function (EF), including problems with working memory (WM) [3–5], inhibitory control [4–7], cognitive flexibility/perseveration [6, 7], and selective and divided attention [7–9]. Most of these deficits have been documented in both controlled neuropsychological studies as well as brain functional magnetic resonance imaging (fMRI) studies showing abnormalities in frontal-striatal circuits [10].

There has been rapid progress in the development of several potentially disease-modifying targeted pharmacological agents, developed through extensive research on the fragile X animal models (*Fmr1* knockouts; for review see [11]). These discoveries paved the way for treatment of the underlying neurobiology of the disorder in humans, including controlled trials of mGluR5 negative modulators, ganaxolone, minocycline, the ampakine CX516, and the cholinergic agonist, donepezil (see review by Berry-Kravis [12]). However, there has been little if any evidence to date that these medications alone can improve behavior or lead to cognitive improvements in this population. As such, effective cognitive and behavioral treatments can fill an important gap in this clinical research space and could represent an empirically supported intervention for families to consider.

Torkel Klingberg and his colleagues at Karolinska Institute showed in a series of studies that WM capacity can be increased with intensive training [13–18]. Stemming from the success of their initial studies, these researchers developed a program that became the basis for the Cogmed computer-based WM training. The training program consists of several different computerized visuospatial memory training tasks, involving the temporary storage (and sometimes manipulation) of sequences in a game format appropriate for the individual’s developmental level. Training success depends in part on visual attention (to encode the sequence of animated figures) and response inhibition (to wait to respond until the sequence is complete), two aspects of executive function that are especially impaired by FXS. Cogmed is likely the most researched cognitive training program, with over 80 original, peer-reviewed research articles (Cogmed Claims and Evidence; https://www.cogmed.com/). Randomized, double-blind, placebo controlled studies documented that Cogmed and other WM training procedures may improve WM and academic achievement, reduce symptoms in children with ADHD, increase auditory attention and WM in preschool children, and improve inattention in daily life (see a review of published studies at https://www.cogmed.com/). However, about the time that the current project was designed and initiated, the efficacy and generalizability of WM training came under considerable scrutiny, with some investigators concluding that “there is little evidence that these programs are suitable as methods of treatment for children with developmental cognitive disorders” and citing limitations of the research, such as the lack of methodological consistency between studies [19–21] and concerns about generalization beyond the training tasks (i.e., far-transfer effects) [20–22]. However, a subsequent "review of reviews" contradicts some of the conclusions [23]. Part of contradictory findings may lie in the theoretical approach with Shipstead arguing that with cognitive training in working memory [21], one should expect to see improvement in general cognitive processes (c.f., [24, 25]). Shipstead et al. nicely detail recommendations for designing studies in order to evaluate the meaningfulness of cognitive training interventions. They include (1) use of multiple measures to assess the broad effect of training on functioning, (2) measure near-transfer effects with valid tools assessing working memory capacity, (3) use control groups that include contact with the experimenters, and (4) use raters blind to study condition for subjective measures. The current project attempted to address concerns raised by Shipstead et al. [21], although this project differs from many of those cited by Shipstead [21] in that our assumption was that working memory training would improve a narrow range of cognitive and behavioral processes associated with impairments in FXS, rather than broad cognitive functioning. We also make the assumption that there is greater plasticity in pediatric-aged participants than in the older adults cited in other commentaries critical of cognitive training [24].

Until our preliminary feasibility study [26], working memory training had not been previously applied to or evaluated in persons with FXS. However, Bennett et al. [27] evaluated and demonstrated the feasibility and preliminary efficacy of Cogmed JM in children with Down syndrome (DS). In the study, 25 children between the ages of 7 and 12 (mean = 9.6 years) and with a mental age between 4 and 7 years (Mean IQ = 65) were randomized into the Cogmed intervention or a waitlist control group. The group completed training in the school setting, facilitated by a special education teaching assistant, 3 times per week for 13 weeks (on average 8.6 h of active training), including feedback and support. The children completed pre- and post-training assessments using the Cogmed-based Index of Improvement (difference in visual memory span from the first 3 training sessions and the highest span achieved during training), non-trained working memory tasks [verbal short-term memory, counting recall, dot matrix, and visual-spatial working memory from the Automated Working Memory Assessment (AWMA)], and parent report of executive functioning. The Down syndrome participants undergoing training had mean span scores of 3.18–3.53 across games at start and mean span scores of 4.09–4.62 at the highest point, with a mean Cogmed Index of Improvement of 14.30. While the wait-listed group of children with DS showed no significant changes, the Cogmed group showed significant improvement on all Cogmed tasks, visual-spatial working memory tasks, and parent ratings of cognitive flexibility and working memory. However, as the authors noted, the study was limited by the wait-list comparison which could not provide blinding or a placebo condition. With the understanding of mixed scientific opinion of the efficacy of these methods, but the conviction that well-standardized and data-driven learning and cognitive training paradigms need to be rigorously studied for FXS, we committed to a trial of Cogmed, the most well-established and investigated program available.

The study aim was to evaluate the efficacy of adaptive Cogmed training (compared to non-adaptive Cogmed) to enhance WM and EF in children and adolescents with FXS in a controlled, randomized, triple-blinded (participant, care provider, outcomes assessor) trial. The primary hypothesis was that individuals with FXS who engage in 25 sessions of adaptive Cogmed training over a period of 5–6 weeks will demonstrate significant improvement on objective, non-trained measures of WM, attention, and inhibitory control compared to those receiving non-adaptive (control) but otherwise identical training.

## Methods

### Participants and randomization

**Fig. 1:**
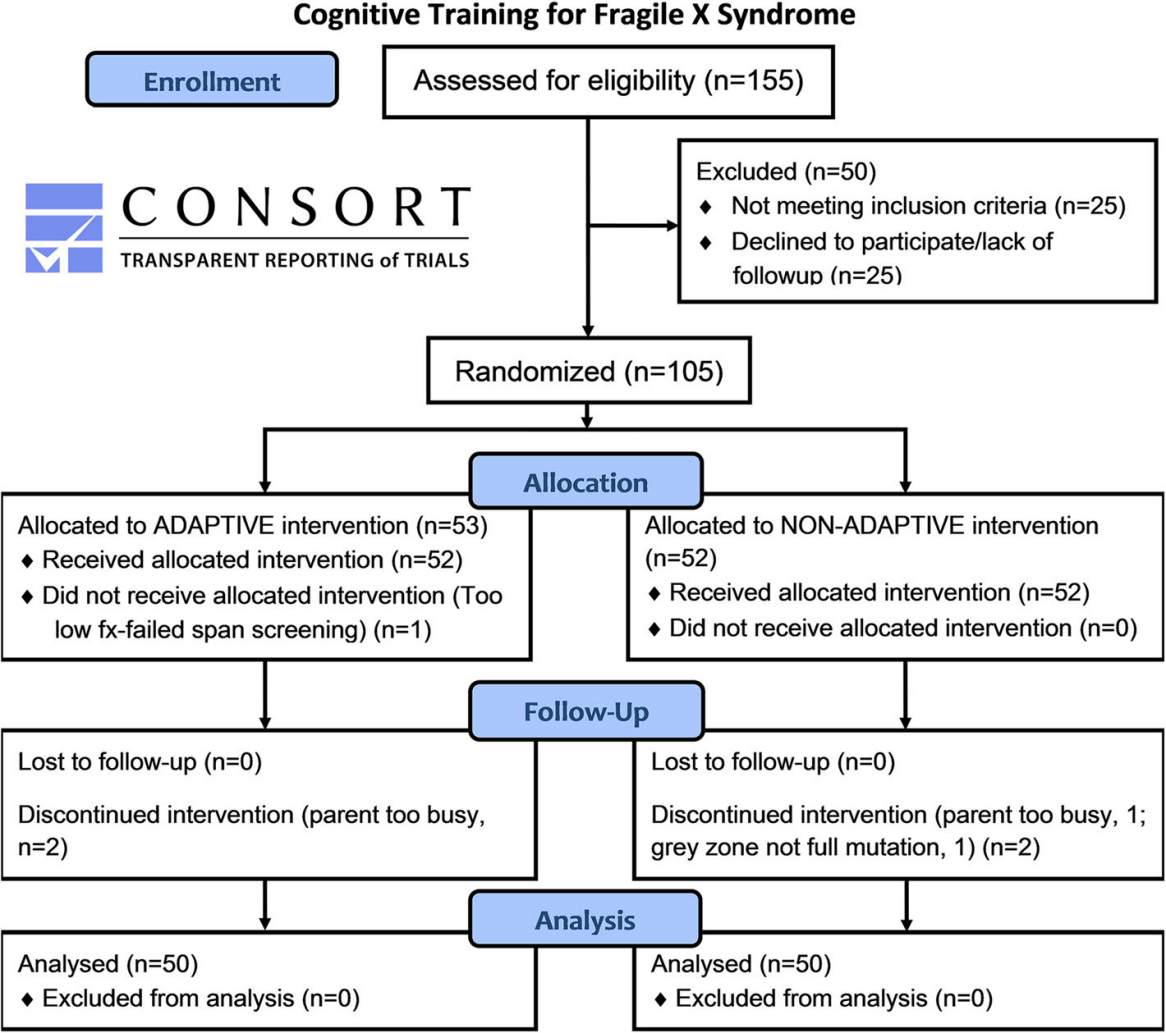
CONSORT diagram showing study screening, enrollment, randomization, retention, and final sample sizes

This study was registered at the clinicaltrials.gov website with identifier NCT02747394. Inclusion criteria were *FMR1* full mutation, as determined by DNA testing, 8–18 years of age, normal or corrected to normal vision and hearing, ability to pass at least some three-span items during a Cogmed training session at baseline, English or Spanish speaking, and parental agreement to maintain adherence to the training schedule and to not alter other treatments during the study. The exclusion criteria were significant brain trauma, previous Cogmed training and significant medical or severe behavioral problems that would interfere with the study. The experimental group completed the usual, publicly available Cogmed adaptive training. For the adaptive version, the difficulty level increases when the participant answers correctly and eases when he or she answers incorrectly and thus memory span is continuously challenged. For the control condition, we chose a non-adaptive version of Cogmed where the span length stays fixed at two throughout all tasks for all sessions and thus memory span is less challenged. Memory span was constrained at 2 span for control training because for many participants with FXS and intellectual disability, 3 or 4 span is the maximum they can achieve at baseline and we wanted to ensure that the span level for the non-adaptive control condition was feasible and would clearly differentiate the groups on level of difficulty. Otherwise these versions are identical. We considered a wait-list control condition but decided against it as this would only allow a comparison of the intervention to no treatment, a less rigorous design [21] lacking specificity (e.g., significant gains in adaptive training compared to wait-list could be attributed to simply playing any computer “game”, the result of the families receiving significant attention from experimenters, or added structure of daily activity). See the Consolidated Standards of Reporting Trials (CONSORT) diagram (Fig. 1) for details of study participants, randomization, and retention. The first participant was enrolled in April 2012 and the last was completed in December 2017. Regarding the 25 participants who were excluded, reasons varied greatly, but the most common were age outside of range, inability to do at least some 3 span items correctly at baseline, and severe behavioral problems.

The final sample of 100 participants, residing in locations throughout the U.S. and Canada (Additional file 1: Figure S1) included 37 females (7 adaptive JM, 5 non-adaptive JM; 11 adaptive RM and 14 non-adaptive RM) and 63 males (25 adaptive JM, 27 non-adaptive JM; 7 adaptive RM, 4 non-adaptive RM). Prior to the study, we determined that for a moderate effect size of 0.6 (standardized difference between groups), the planned analysis of covariance (ANCOVA) with *n* = 100 participants would have power between 87% and 93% when the correlation between baseline and six-week measurements ranges from 0.25 to 0.5 (two-sided test at level alpha = 0.05). The expected effect size of 0.6 is consistent with those reported in prior Cogmed trials, including those involving an intellectually disabled population (DS [27]). Assignment to RM (school-age version suitable for higher functioning participants; see details below) vs. JM (preschool version suitable for lower functioning participants; see details below) was determined based on screening, baseline cognitive testing, clinical judgment, and ability to understand and complete RM games. Participants who were high functioning enough and could complete at least 11 of 13 RM games at baseline during the first visit were assigned to RM. In the adaptive group, all 50 participants (100%) were adherent to treatment (20–25 days of training, mean = 24.22 days) and in the non-adaptive group 49/50 (98%) were adherent (1 participant with 18 training days, mean = 24.22 days).

#### The Cogmed training program

Cogmed JM, intended for preschool age children and based on an amusement park theme, consists of seven different computerized visuospatial memory training tasks. Each task involves the temporary storage and, for some tasks, manipulation of visuospatial sequences. For example, bumper cars that move around the screen and light up one at a time are recalled in order by the participant who either clicks with the computer mouse or touches the cars on the screen. Four of the seven tasks involve only the storage of visual information (pool, hotel, rollercoaster, twister), two involve both manipulating and storage of visual information (Ferris wheel, bumper cars), and one involves the storage of visual and auditory information (wheel of animals). In each training session, participants completed three of the seven training activities. Sessions typically require 15 min. Cogmed RM is intended for school-age children above 7 years, lasts about 30 min per session, and includes 14 different tasks centered on a robot theme. The tasks are more complex and challenging than JM, involving rotating displays, moving targets, reverse sequence tasks, numeric information to recall, and delayed responses. Each version of Cogmed (adaptive JM and RM) provides an Index of Improvement metric, defined as the difference in average visual memory span from the first 3 training sessions (across tasks) and the current highest average span achieved during training.

#### Study procedures

After primary inclusion criteria were satisfied, an examiner traveled to the home to consent/assent and collect baseline data. (Examiners and Cogmed coaches were different staff members so that the examiners were kept blind to treatment condition.) During the first visit, the examiner completed baseline assessments (Time 1), worked with participants briefly on the Cogmed RM game “asteroids” to ensure capacity for 3 span training, and worked with the parents on how to support participants during training sessions (e.g., positive reinforcement, visual schedules, reward chart) and to ensure that the training location was optimal. Following completion of baseline assessments, participants were randomized (using a random number generator) equally to one of two groups: (1) adaptive Cogmed or (2) non-adaptive Cogmed (control). The group assignment was concealed from the staff conducting the study. All participants maintaining active enrollment in the study had no change in treatment (pharmacological, behavioral) during the course of the training period. Parent training aides and teacher raters were kept blind to treatment group, and parents were asked to inform teachers that their children are in a research study and to avoid any mention of treatment. The examiners explained to the caregivers that the study is comparing two levels of training intensity (higher memory load vs. lower memory load) and did not use the words “placebo,” “adaptive,” or “non-adaptive.” However, we should note that because parents served as the training aides, it is possible that some surmised that their child was in the higher or lower memory load group, despite the fact that they were not exposed to the other training load condition. The parent training aide was explicitly told not to help their child during training (e.g., instructing them where to look or what targets to press), although multiple methods of encouragement and reinforcement were allowed. The two groups completed 5–6 weeks of training 5 days a week to achieve the goal of 25 total training sessions. After training (and within 1 week of completion), the examiner returned to the home to complete Time 2 assessments using the same outcome measures. To examine whether improvements related to Cogmed, if present, are maintained after training stops, examiners also completed a follow-up assessment 3 months later (Time 3). The same examiner completed all assessments for each participant. All participants completed Cogmed training with their caregiver/parent as the training aide, and the same training aide/parent was present for all training sessions. All participants received a weekly call by a staff member who completed coach training per Cogmed guidelines. Coaches focused on working with parent training aides on maintaining motivation (e.g., by making adjustments to methods and type of reinforcement and utilizing a picture schedule showing a timeline of training sessions which could be crossed off), dealing with any technological problems, managing challenging behaviors, minimizing distractions in the environment, encouraging breaks in training when needed, and adjusting time of day for training as needed to maximize focus and compliance.

#### Working memory outcome measures

The *Leiter*-*Revised* (*Leiter*-*R*) *Spatial Memory* subtest was used as one of two objective measurements of visual WM. The Leiter-R is sensitive to visual WM impairments in children/adolescents with FXS [3, 28]. For this study, rather than counting one point per correct trial, we calculated the total number of correctly recalled objects, allowing for greater range. The *Stanford Binet 5 Block Span* subtest requires the participant to watch the examiner tap blocks in a particular order and then recall the pattern by tapping the blocks in the same order. For this study, we added several additional easier items, also to allow greater range. The composite (average) of the Spatial Memory and Block Span scores was used as the primary and pre-specified outcome measure for this trial. To measure auditory WM, the *Wechsler Intelligence Scale for Children*, *Fourth Edition (WISC-IV) Digit Span* subtest was used.

#### Executive Function Outcome Measures

The *Kiddie Test of Attentional Performance* (*KiTAP* [29]) is a computer-administered measure of EF designed around an enchanted castle theme. A panel of seven scores from four subtests was identified as feasible; lacked ceiling, basal, or learning effects; exhibited an acceptable range; had good reproducibility; and correlated significantly with ratings for hyperactivity and/or attention in our prior studies with FXS participants [29].

#### Behavioral Ratings of Attention and Executive Function

The *Conners Third Edition* (*Conners 3* [30]) is a multi-informant assessment of ADHD-related behavior that takes into account home, social, and school settings. The *Behavior Rating of Executive Function* (*BRIEF* [31]) is a standardized measure of behaviors related to EF for children. Both caregivers and teachers provided Conners and BRIEF ratings, using versions of the scales appropriate for the participant’s mental age. The Inattention and Hyperactivity/Impulsivity subscales of the Conners and the WM and Global Executive Composite scales of the BRIEF were used. Teacher ratings were not possible for participants who had assessments during summer months or for participants who were home-schooled.

#### Statistical analyses

The pre-specified efficacy analysis for the WM composite score was analysis of covariance (ANCOVA) of outcome measures after 5–6 weeks of treatment adjusted for baseline measures. Analyses of secondary measures (BRIEF, Connors, KiTAP, and Digit Span) were also based on the ANCOVA model, adjusted for baseline measures. Secondary analyses (of both primary and secondary outcomes) to compare scores at baseline, post-training, and 3-month follow-up were based on linear mixed effects model. Post hoc analyses were also performed to examine the two versions of Cogmed separately (JM and RM). Additional post hoc descriptive analyses compared “improved” and “not improved” participants within adaptive and non-adaptive control groups, using twice the average improvement over baseline (IOB) with respect to the primary outcome (visual working memory composite) as the cut-point to divide these groups. All tests, except for primary efficacy, were at a level of 0.05 and analyses were implemented in SAS® software Version 9.4. Also, we examined the test-retest reliability of the outcome measures in this study sample in order to determine the stability of these measurements in this population and to inform future research. This was done using the intraclass correlation coefficient (ICC) with data collected at the post-training visit and the 3-month follow-up for all subjects.

## Results

### Participant characteristics at baseline

The adaptive and non-adaptive participant groups did not differ significantly by race, ethnicity, parent education level, household income, parent marital status, psychoactive medication use, age, abbreviated IQ, mental age, or total number of Cogmed training days (Table 1, descriptive statistics). Participants in the adaptive group spent about 3 min more active training time per day than those in the non-adaptive control group (*p* < .05). Active training time was used as a covariate in analyses below.

**Table 1:** Participant and parent characteristics by adaptive vs. non-adaptive control group

Variable	Category	Non-adaptive		Adaptive		*p* value
		N	%	N	%	
Participant race^a^	Caucasian	38	76.00	41	82.00	0.461^a^
Participant ethnicity^a^	Hispanic or Latino	11	22.00	7	14.00	0.298^a^
Parent 1 education (training aide) ^a^	Bachelor’s or above	33	66.00	33	67.35	0.545^a^
Parent 2 education^a^	Bachelor’s or above	34	70.83	22	47.83	0.826^a^
Household income	$75 K+	34	69.39	29	59.18	0.237
	<$75 K	10	20.40	15	30.61	
	Prefer not to report	5	10.20	5	10.20	
Participant psychoactive medications	SSRI/SNRI	21	42.00	21	42.00	
	Stimulant	14	28.00	22	44.00	
	Antipsychotic	3	6.00	6	12.00	
	Glutamatergic	3	6.00	1	2.00	
	GABAergic	1	2.00	0	0.00	
	Alpha agonist	2	4.00	2	4.00	
	Any	31	62.00	32	64.00	0.836
Parent marital status	Married	46	92.00	46	93.88	0.511
Cogmed training platform	PC with mouse	13	26.00	12	24.00	0.817
	Tablet	37	74.00	38	76.00	
		Mean	SD	Mean	SD	*p* value
Participant age		12.26	3.04	13.00	3.11	0.232
Abbreviated IQ (SB 5)		64.79	15.64	64.42	17.73	0.914
Mental age equivalent (years; SB 5)		6.98	1.90	7.31	3.91	0.323
Cogmed sessions per week		4.46	0.83	4.38	1.09	0.710
Total training days		24.22	1.47	24.22	1.79	1.000
Active training time per day (min)		18.01	5.98	21.08	8.10	0.033

*p* values reflect differences utilizing all categories

^a^Not all categories are shown to conserve table space

### Cogmed working memory training Progress

**Fig. 2:**
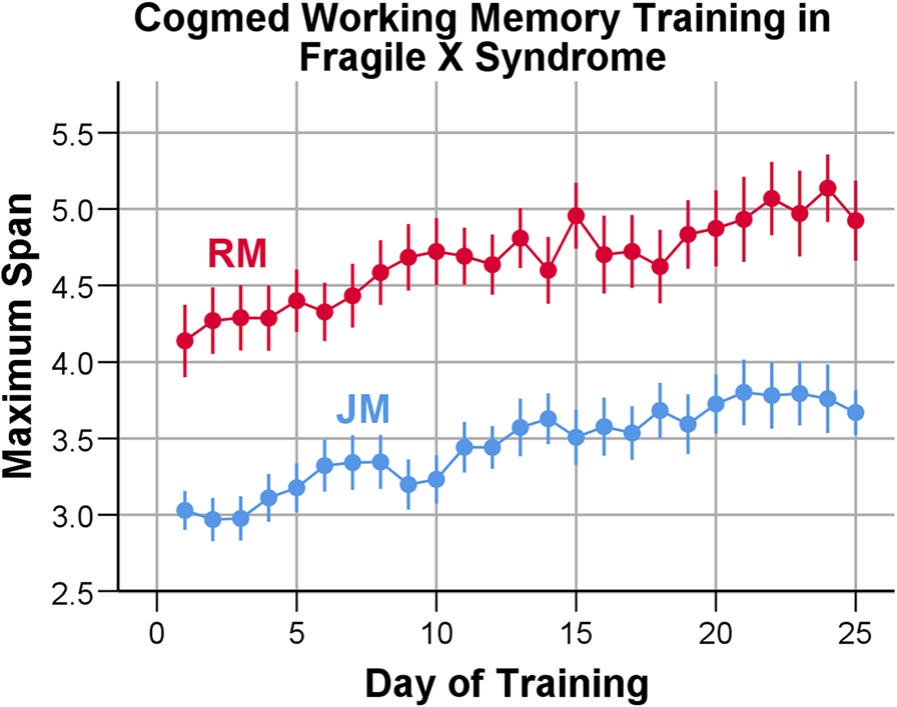
Maximum average working memory span length (index) by training day in participants with fragile X syndrome completing adaptive Cogmed JM (*N* = 32; blue) or RM (*N* = 18; red) training. JM and RM participants showed significant increases in memory span between Start Index and Maximum Index (JM, paired *t* (31) = 13.45, *p* < .0001, Cohen’s *d* = 2.38; RM, paired *t* (17) = 17.74, *p* < .0001; Cohen’s *d* = 2.62). Error bars show 95% confidence intervals

As can be seen in Fig. 2, Cogmed JM adaptive participants started training with a maximum span length of just over 3.0 and improved modestly and steadily to a maximum span of about 3.8 near the end of training (mean Index of Improvement = 14.81). A paired-samples *t* test showed a significant increase in memory span between Start Index and Maximum Index, *t* (31) = 13.45, *p* < .0001; Cohen’s *d* = 2.38. Cogmed RM adaptive participants began at an approximately 4.2 span length and finished at a maximum of approximately 5.2 near the end of training (mean Index of Improvement = 21.89). For the RM group, a paired-samples *t* test also documented the significant increase in memory span, *t* (17) = 17.74, *p* < .0001; Cohen’s *d* = 2.62.

### Visual working memory (primary outcome)

For the WM composite, the average score post-training relative to baseline was slightly higher for adaptive compared to non-adaptive groups, although the baseline-adjusted group difference post-training was not statistically significant (Table 2). The linear effects mixed model showed that in both adaptive and non-adaptive control training groups, primary WM outcomes were significantly improved post-training compared to baseline on both tests (Table 3). Furthermore, improvements at 3-month follow-up were maintained and similar to levels seen at the completion of training (Table 3, 3-month follow-up vs. post-training).

**Table 2:** Primary and secondary outcomes analyses (by treatment condition)^a^

Variables	Non-adaptive control						Adaptive						*p* value
	Baseline			Post-training			Baseline			Post-training			
	*N*	Mean	SD	*N*	Mean	SD	*N*	Mean	SD	*N*	Mean	SD	
(A) Primary measures													
Leiter-Revised Spatial Memory	50	21.44	10.00	49	22.94	9.87	50	20.44	14.09	49	24.27	13.51	
Stanford Binet-5 Block Span	50	10.66	4.65	49	11.65	4.86	50	10.20	5.40	50	11.92	5.75	
Visual Working Memory Composite (mean)	50	16.05	6.62	49	17.30	6.71	50	15.32	9.15	49	18.19	8.98	0.533
(B) Secondary measures													
Digit Span (auditory working memory)	49	8.02	4.02	48	8.54	3.82	49	7.51	4.69	49	8.53	5.12	0.888
BRIEF													
Parent—Working Memory	48	21.48	3.95	47	20.53	4.23	49	22.12	3.84	49	21.49	4.17	0.082
Teacher—Working Memory	28	21.57	5.65	23	20.83	5.59	31	23.13	5.26	30	21.70	5.84	0.313
Parent—Global Executive Composite	46	151.61	21.82	46	144.41	24.83	45	151.38	27.63	47	148.13	25.83	0.022
Teacher—Global Executive Composite	27	143.30	32.06	22	142.36	34.04	29	143.79	32.28	30	137.83	33.75	0.182
Conners													
Parent—Inattention	47	17.98	5.88	46	16.67	6.72	49	18.80	6.32	49	17.53	6.95	0.527
Teacher—Inattention	28	18.32	9.11	25	16.20	8.03	31	18.07	8.30	30	15.80	8.52	0.499
Parent—Hyperactivity/Impulsivity	47	18.23	10.88	46	16.94	12.02	49	18.55	11.71	49	18.45	12.54	0.319
Teacher—Hyperactivity/Impulsivity	28	25.50	14.76	11	29.73	16.90	31	22.90	16.46	14	22.64	17.47	0.820
KiTAP													
Distractibility Errors	43	16.84	11.02	40	14.83	11.58	40	15.35	10.27	41	13.29	11.38	0.920
Alertness SD of RT	48	240.18	266.98	45	230.40	206.09	45	344.76	294.35	47	309.77	286.70	0.189
Flexibility False Alarms	42	8.05	4.27	38	7.13	3.81	37	9.24	3.87	38	7.53	4.50	0.985
Go NoGo False Alarms	47	4.34	4.84	44	4.93	5.39	41	4.00	4.96	46	5.35	5.08	0.153

*BRIEF* Behavior Rating of Executive Function, *SD* standard deviation, *RT* reaction time, *KiTAP* Kiddie Test of Attentional Performance, *GEC* Global Executive Composite

^a^Results adjusted for active training time

**Table 3:** Linear mixed effect model comparison of outcomes over time (combined groups)

Outcome	Comparison	Estimates		
		Est.	SD	*p* value
(A) Primary outcomes				
Leiter-Revised Spatial Memory	PT vs. BL	2.62	0.85	0.003
	FU vs. BL	2.21	0.89	0.014
	FU vs. PT	− 0.41	0.90	0.650
Stanford Binet-5 Block Span	PT vs. BL	1.41	0.25	< .0001
	FU vs. BL	1.36	0.26	< .0001
	FU vs. PT	− 0.05	0.26	0.856
(B) Secondary outcomes				
Digit Span	PT vs. BL	0.85	0.20	< .0001
	FU vs. BL	1.30	0.21	< .0001
	FU vs. PT	0.45	0.21	0.029
BRIEF				
Parent—Working Memory	PT vs. BL	− 0.87	0.29	0.003
	FU vs. BL	− 0.74	0.30	0.015
	FU vs. PT	0.13	0.30	0.668
Teacher—Working Memory	PT vs. BL	− 1.16	0.41	0.006
	FU vs. BL	− 1.90	0.54	0.001
	FU vs. PT	− 0.74	0.54	0.172
Parent—GEC	PT vs. BL	− 5.65	1.46	< .0001
	FU vs. BL	− 4.04	1.50	0.008
	FU vs. PT	1.62	1.49	0.279
Teacher—GEC	PT vs. BL	− 4.93	2.45	0.048
	FU vs. BL	− 9.20	3.21	0.006
	FU vs. PT	− 4.27	3.19	0.185
Conners				
Parent—Inattention	PT vs. BL	− 1.38	0.44	0.002
	FU vs. BL	− 0.80	0.45	0.080
	FU vs. PT	0.58	0.46	0.203
Teacher—Inattention	PT vs. BL	− 2.03	0.70	0.005
	FU vs. BL	− 1.63	0.98	0.100
	FU vs. PT	0.40	0.97	0.678
Parent—Hyperactivity/Impulsivity	PT vs. BL	− 0.76	0.56	0.179
	FU vs. BL	0.16	0.58	0.787
	FU vs. PT	0.91	0.58	0.118
Teacher—Hyperactivity/Impulsivity	PT vs. BL	− 0.69	1.02	0.498
	FU vs. BL	− 1.35	1.40	0.338
	FU vs. PT	− 0.65	1.39	0.639
KiTAP				
Distractibility Errors	PT vs. BL	− 2.14	0.92	0.021
	FU vs. BL	− 1.64	1.05	0.119
	FU vs. PT	0.50	1.04	0.631
Alertness SD of RT	PT vs. BL	− 20.11	24.98	0.422
	FU vs. BL	19.57	28.10	0.487
	FU vs. PT	39.67	27.94	0.158
Flexibility False Alarms	PT vs. BL	− 1.37	0.39	0.001
	FU vs. BL	− 1.28	0.44	0.004
	FU vs. PT	0.10	0.43	0.823
Go NoGo False Alarms	PT vs. BL	1.02	0.46	0.030
	FU vs. BL	0.62	0.53	0.240
	FU vs. PT	− 0.39	0.52	0.453

*BRIEF* Behavior Rating of Executive Function, *PT* post-training, *FU* follow-up, *BL* baseline, *SD* standard deviation, *RT* reaction time, *KiTAP* Kiddie Test of Attentional Performance, *GEC* Global Executive Composite

### Auditory working memory (digit span)

Digit Span increased significantly in both treatment groups after training (Table 3); however, the adaptive group did not improve significantly more than the non-adaptive group (Table 2). The linear effects model showed further significant improvement for both groups after the 3-month follow-up for this measure.

### Attention, inhibitory control and cognitive flexibility (KiTAP)

For the KiTAP, overall participants made significantly fewer false alarms on the Flexibility test at post-training and follow-up compared to baseline, and significantly fewer errors on the Distractibility test after training (Table 3). Participants receiving adaptive training did not significantly outperform those in the non-adaptive control group after treatment on any KiTAP test (Table 2).

### Parent and teacher ratings of behavior and executive function (Conners, BRIEF)

Parents and teachers reported significant reductions in problems of attention, WM, and global EF between baseline and post-treatment (Table 3), but no significant differences in improvement were observed between treatment conditions (Table 2). Parents of children in the adaptive group (compared to non-adaptive) reported significantly less improvement on the global executive score of the BRIEF. However, neither parents nor teachers reported significant changes in hyperactive/impulsive behaviors during the treatment period for either group.

### Post hoc analysis: examination of Cogmed JM and RM training

Participant and demographic characteristics did not differ between adaptive (A) and non-adaptive (NA) groups within JM (Additional file 2: Table S1) or RM (Additional file 3: Table S2), except for average active training time per day similar to the overall cohort (Table 1). Between JM an RM cohorts, participants in RM had higher mean IQ (NA 76.8, A 79.6) than participants in JM (NA 58.3, A 55.9) and higher mental age by ~ 3.5 years. Although total training days were similar between JM and RM groups, as expected the active training time per day (min) was greater in RM (NA 24.8, A 30.8) than JM (NA 14.2, A 15.6). The JM group had a higher proportion of Hispanic or Latino families and lower proportion of higher education (Bachelor’s or above).

**Fig. 3:**
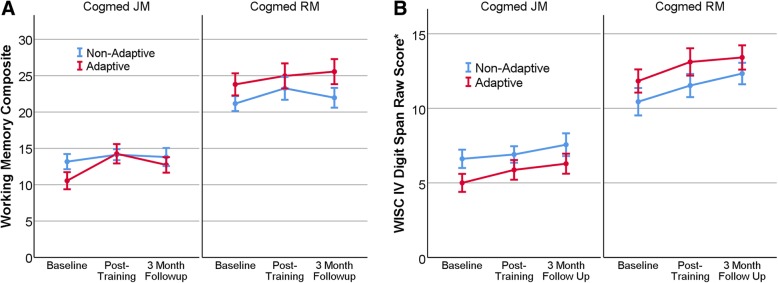
Composite visual memory score (**a**; primary outcome; mean of Leiter Spatial Span and SB-5 Block Span) and **b** auditory working memory score (WISC IV Digit Span) at baseline, post-training, and at 3-month follow-up (no training), by Cogmed version (RM, higher functioning, *N* = 36; JM, lower functioning, *N* = 64) and by treatment condition (adaptive, *N* = 50 vs. non-adaptive control, *N* = 50) in children and adolescents with fragile X syndrome. Adaptive and non-adaptive groups combined demonstrated significant gains in visual and auditory working memory after training (*p* < .0001), and significant gains in auditory working memory also continued after 3 months of no treatment (*p* < .05). Post hoc analyses demonstrated significant improvement in the JM adaptive compared to non-adaptive groups from baseline to post-training (**a**), but no relative difference in efficacy for the RM version. Scores are raw observed scores ± SEM

Separate models comparing adaptive and non-adaptive control training for JM and RM versions revealed different results with regard to the primary outcome measure, the visual memory composite. For JM, the adaptive group significantly outperformed the control group for this near-transfer measure (Fig. 3); however, relative improvement of adaptive training did not generalize to other domains of measurement for this subgroup (Additional file 4: Table S3). There was a nearly (*p* = .053) significant difference on the KiTAP Alertness test for JM, however the control group reduced variability in reaction time after training more so than the adaptive group. RM training yielded no significant benefits of adaptive over non-adaptive control training on any measure. For RM, the non-adaptive group had a larger decrease in parent-reported EF problems than the control group.

### Post hoc analysis: examination of “improved” vs. “not improved” participants

For the adaptive group, the average improvement over baseline (IOB) with respect to the primary outcome (visual working memory composite) was about 2 points. Thus, we defined “improved” as individuals whose improvement was at least twice the average IOB (i.e., improvement of 4 points or more) vs. “not improved” as IOB < 4. Results are summarized in Additional file 5: Table S4. Participant and parent characteristics, including IQ, were not different between those who improved (*n* = 15) and did not improve (*n* = 26). However, the group that improved on average had one more training day (~ 25 vs. 24 days) during the study. A similar analysis was conducted for the control group (Additional file 6: Table S5). Overall, there was no difference in characteristics between the groups that improved (*n* = 11) and did not improve (*n* = 27), except that the group that improved was older (13.6 vs. 11.7-years old). We caution that reader that these ad-hoc supplemental analyses are descriptive and exploratory.

### 3-month test-retest reliability of outcome measures

The test-retest intraclass correlation coefficients for the outcome measures are shown in Additional file 7: Table S6. With the exception of the measure of Alertness on the KiTAP test, stability during the 3-month follow-up was good to excellent.

## Discussion

The results of this controlled trial of working memory training in children and adolescents with FXS showed that objectively-evaluated WM and some domains of EF, as well as parent- and teacher-reported attention and EF-related behaviors significantly improved during the treatment period, with many changes maintained at follow-up after 3 months without training. However, contrary to the primary study hypothesis, degree of improvement between adaptive treatment and control groups did not differ significantly, showing that progressively challenging the WM system by expanding span length (which adaptive Cogmed participants were able to achieve) did not provide added benefit in the full range of individuals with this disorder.

A potential explanation for why improvements were seen in both the non-adaptive control and adaptive conditions is that the study is more an assessment of dose of training, with the non-adaptive a “low level” training, rather than a comparison of active versus placebo conditions. Both conditions require active use of WM processes, and other studies have demonstrated improvements on objective outcome measures in the non-adaptive condition [32, 33]. It may be that the non-adaptive condition could have greater impact on individuals with a relatively lower baseline level of working memory functioning, for whom a typical span length might be closer to 2–4 items capacity. The non-adaptive condition level is set at a span length of 2 items, which might be already close to the maximum of what our participants with intellectual disability were capable of and therefore be quite challenging. Thus, detecting a significant difference between the adaptive and non-adaptive condition could be difficult given that this is more of a comparison between a low and high dose of training, and in this regard a larger sample may be necessary to detect a difference between two active treatments.

It is possible that the improvements seen in both groups (adaptive and non-adaptive) reflect practice or rater expectancy effects rather than changes associated with training. In retrospect, it may have been helpful to include a wait-list control group (especially for comparison to non-adaptive control), although this type of design has limitations as well, namely the comparison of any treatment to none (lack of specificity) and contamination by uncontrolled placebo and expectancy effects which are well known in trials of patients with FXS and other neurodevelopmental disorders, particularly on the subjective measures (i.e., rating scales). From the perspective of the performance measures, there is little if any evidence from prior studies that cognitive scores of individuals with FXS improve significantly over a period of weeks or months without intervention. For example, in our work on the NIH Toolbox Cognitive Battery, 26 individuals with intellectual disability (ID) (including 10 with FXS) of approximately the same chronological and mental ages (mean = 16 years and 5 years, respectively) completed the List Sort WM test at baseline and after 4 weeks of no intervention and did not significantly improve (*p* = .40, Cohen’s *d* = .17) [7]. There also were no other significant improvements over that time span on any of the other 6 subtests. In the prior work on the KiTAP in 27 young adults with FXS retested after 2–4 weeks [29], there were no significant changes in performance on the same 4 key measures utilized in the present study. Finally, in other published work, wait-list controls in the previously-described study of the efficacy of Cogmed JM in children with DS showed no significant improvement on performance measures of verbal or visual-spatial short-term memory or WM [27]. In the DS study, participants had almost exactly the same gains on JM as our FXS adaptive JM participants (Index of Improvement ~ 14). We recognize that the data collected in these other studies were collected under circumstances that differ from the trial presented here. However, we felt that a comparison of test-retest data from individuals with FXS and DS who are not receiving treatment or intervention would be useful in interpreting the present cognitive training results.

Placebo effects could explain the observed improvements, and prior work suggests they may be seen even on performance measures in persons with ID [34]. Thus, it is important to try to evaluate the degree to which improvements seen in the present study are due to the treatment itself vs. various other factors that may lead to improvement such as attention to the participant, the caregiver’s expectations of the treatment, and the participant’s own expectancy to improve through training. Three results of the study suggest that placebo effects are unlikely to fully explain the improvements. First, many improvements during the course of training were maintained after 3 months of no training, and in no case did performance return to pre-training baseline. Second, teachers reported significant improvements in WM, global EF, and attention in another environment (school) across both groups, and they were kept unaware of the purpose of the study. And third, improvements were not observed in all areas, suggesting that effects may be more related to attention and EF. In particular, no significant changes were seen in hyperactive/impulsive behavior and there was a significant *increase* in false alarms on the KiTAP Go NoGo test after training. Another potential explanation for the improvements seen in both adaptive and non-adaptive groups, is that a more general process of regularly engaging in tasks that require sustained attention, response inhibition, remaining seated, following instructions, and pressing on and completing a task over time, while guided by a supportive training aide (parent, in this case), is therapeutic for individuals with FXS, who have well-documented problems in all of these areas. Also, as stated by Chacko and colleagues who also observed gains associated with non-adaptive training [32], supportive counseling and coaching of parents (a primary role of the Cogmed coach) can contribute to reduced ADHD symptoms (e.g., Sonuga-Barke et al. [35]). In this regard, a non-therapeutic computer game with matched active time and duration may have been helpful as a contrast condition. If the “general structured training” interpretation of the observed gains is correct, there may be a range of parent-guided one-on-one cognitive and/or behavioral training approaches that can be beneficial to children with FXS. As such these results may have implications for the design of future treatment studies.

Given the pattern of results in the study, a natural question is: Is Cogmed an empirically validated treatment for children and adolescents with FXS, forming the basis for treatment recommendation? Unfortunately, without a clear separation of the adaptive from non-adaptive control groups (the a priori hypothesis) or separation of Cogmed training from another inactive control, it is not possible to draw this conclusion, despite the possibility that both levels of training may have contributed to the observed changes. Also, for the primary outcome measures, the effect sizes were modest, with Cohen’s *d* in the range of .21–.31 for the adaptive group. For the teacher and parent ratings, effect sizes were in the range of .20–.27. Although certainly a challenge, future studies examining cognitive training effects in ID populations such as FXS will need to carefully consider and select treatment comparison conditions to ensure the absence of an active control. Future analyses will help to identify subgroups with the best response to treatment, and in those cases effect sizes are likely to be larger. Inter-individual differences (e.g., quality of training, baseline capacity, co-morbidity, training environment, characteristics of training aide) may be associated with training outcomes and transfer effects and may be particularly critical in populations with ID [36]. If any of these key factors do moderate efficacy, the results may help to link training to outcomes and will help to identify those families that are the best candidates for this type of intervention. In the current study, baseline participant and family demographic factors did not differ in those who demonstrated substantial improvement on the primary outcome measure; although, it is of potential interest that the “improved” and “not improved” adaptive groups showed a modest but significant difference in the number of training days completed. Finally, as no other controlled trials of cognitive or behavioral interventions for FXS are available, it is difficult to determine whether alternative interventions have stronger effects.

The limitations of the study include the lack of a secondary comparison group (wait-list control or clearly inactive control), variation in the control of the training environment (homes) and training aides (parents of varying skill, nature of parent-child relationship), a sub-optimal sample size for examining teacher-reported results, and a lack of caregiver data on satisfaction with the treatment approach. The males in the study were on average somewhat higher functioning (mean abbreviated IQ = 56.8) than the overall population of males with FXS, and as such, it is important to recognize that lower functioning individuals may not be able to engage in the training. Another possible limitation of Cogmed, and likely other forms of cognitive training, is that it cannot be maintained indefinitely while providing adequate interest and engagement. Other forms of empirically validated treatment may need to be integrated with the individual’s repertoire of educational remediation and therapies to maintain engagement and provide more generalized and sustained benefits. Despite these limitations, the project provides strong evidence that well-controlled, home-based cognitive/behavioral trials can be completed with reliable and valid endpoints in individuals with FXS, suggesting similar success can be achieved in other populations with ID. The unusually low drop-out rate (3/105 participants enrolled) lends support to the feasibility and acceptability for this intervention with this population; although, we recognize that the participating families were relatively high-functioning (as reflected by marital status and income); as such, it may be that those from more disadvantaged backgrounds or single-parent households would have lower adherence rates.

A next step in this research program would be to evaluate whether cognitive training such as Cogmed or other attention training programs [37] and potentially beneficial effects on attention and EF can be accelerated by targeted pharmacological treatment. This combined approach fits with a model whereby targeted treatment (e.g., to restore more normal synaptic transmission, perhaps through improved balance of inhibitory and excitatory brain mechanisms) provides individuals with FXS with an enhanced potential to learn and gain function through intensive cognitive or behavioral therapy.

## Conclusions

This study provides evidence that children and adolescents with FXS can engage and make progress in an intensive web-based working memory training program, Cogmed, over a period of 5–6 weeks. However, the primary hypothesis that participants completing the publically available adaptive training versions of the program will make significantly greater gains in standardized measures of working memory than those completing a non-adaptive “control” version was not confirmed; in fact both groups improved significantly on a variety of metrics. Future studies with a non-active control condition or larger sample sizes may be needed to determine whether Cogmed itself, rather than the potentially broader therapeutic aspects of the intervention, is effective for this population.

## Additional files

Additional file 1: **Figure S1**. Map showing participant home locations. (TIF 841 kb)

Additional file 2: **Table S1**. Cogmed JM participant and parent characteristics by adaptive vs. non-adaptive control groups. (XLSX 10 kb)

Additional file 3: **Table S2**. Cogmed RM participant and parent characteristics by adaptive vs. non-adaptive control groups. (XLSX 10 kb)

Additional file 4: **Table S3**. Post hoc analyses of primary and secondary measures stratified by Cogmed version (A) JM and (B) RM*. (DOCX 27 kb)

Additional file 5: **Table S4**. Participant and parent characteristics by “improved”* vs. “non-improved” within the adaptive group. (XLSX 11 kb)

Additional file 6: **Table S5**. Participant and parent characteristics by “improved”* vs. “non-improved” within the non-adaptive group. (XLSX 10 kb)

Additional file 7: **Table S6**. Test-retest reliability (intraclass correlation) of outcome measures used in the study (during 3-month follow-up period without training). (DOCX 14 kb)

## Abbreviations

ADHD: Attention-deficit hyperactivity disorder
ANCOVA: Analysis of covariance
BRIEF: Behavior Rating of Executive Function
CONSORT: Consolidated Standards of Reporting Trials
EF: Executive function
*FMR1*: Fragile X mental retardation 1
fMRI: Functional magnetic resonance imaging
FXS: Fragile X syndrome
ICC: Intraclass correlation coefficient
ID: Intellectual disability
IQ: Intelligence quotient
KiTAP: Kiddie Test of Attentional Performance
mGluR5: Metabotropic glutamate receptor 5
MIND: Medical Investigation of Neurodevelopmental Disorders
WM: Working memory
