# Supplementary figures and images for: Cognitive training for children and adolescents with fragile X syndrome: a randomized controlled trial of Cogmed
Source: J Neurodev Disord. 2019 Apr 15;11:4. doi: 10.1186/s11689-019-9264-2 (PMC6463634; doi:10.1186/s11689-019-9264-2)

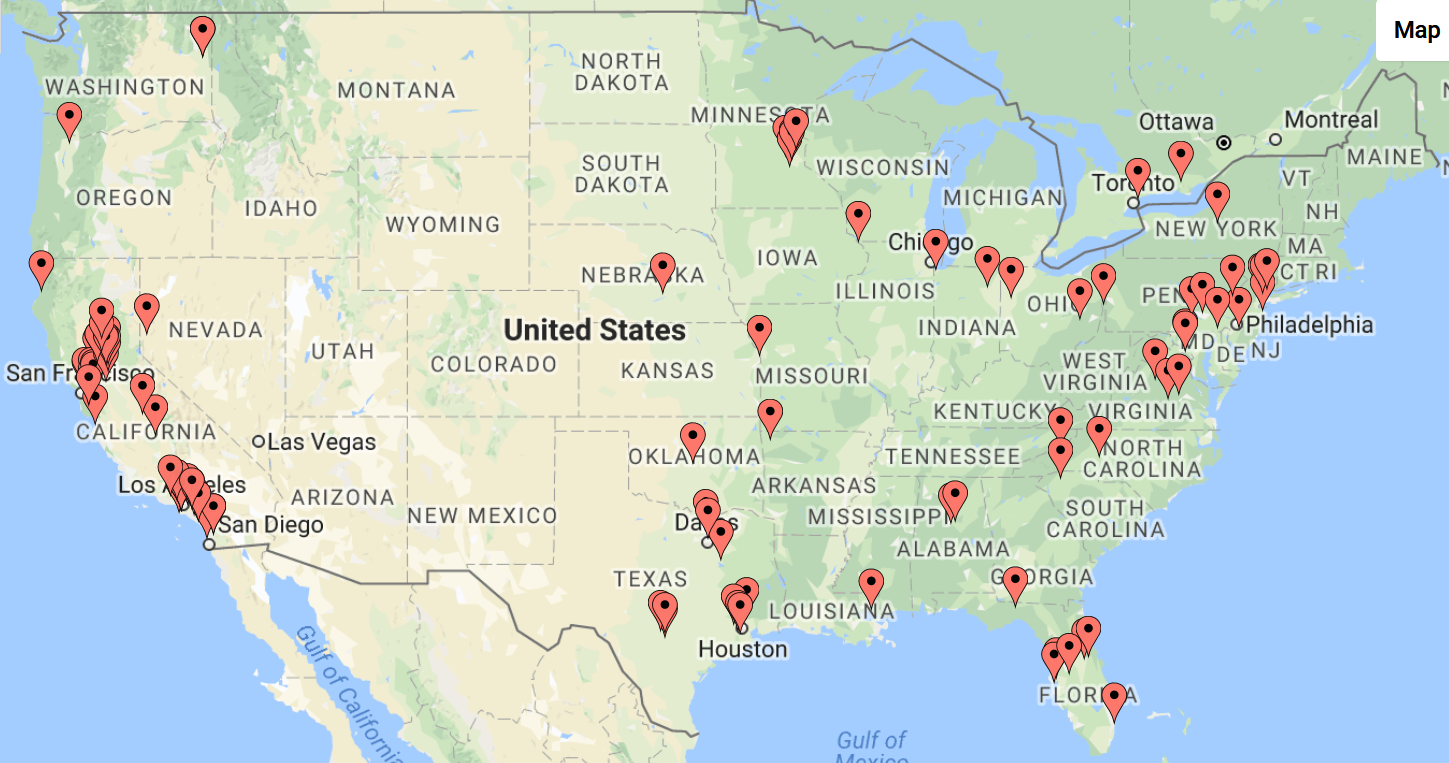

Supplement: Supplementary file 1 — Figure S1. Map showing participant home locations. (TIF 841 kb) [file 11689_2019_9264_MOESM1_ESM.tif]
