# Supplementary material for: Cognitive training for children and adolescents with fragile X syndrome: a randomized controlled trial of Cogmed
Source: J Neurodev Disord. 2019 Apr 15;11:4. doi: 10.1186/s11689-019-9264-2 (PMC6463634; doi:10.1186/s11689-019-9264-2)
Supplement: Supplementary file 4 — Table S3. Post hoc analyses of primary and secondary measures stratified by Cogmed version (A) JM and (B) RM*. (DOCX 27 kb) [file 11689_2019_9264_MOESM4_ESM.docx]

| **Table S3. Post hoc analyses of primary and secondary measures stratified by Cogmed version (A) JM and (B) RM*** | | | | | | | | | | | | |  |
| --- | --- | --- | --- | --- | --- | --- | --- | --- | --- | --- | --- | --- | --- |
|  | **Non-Adaptive** | | | | | | **Adaptive** | | | | | | **P-value** |
| **(A) Cogmed version JM** | **Baseline** | | | **Post-training** | | | **Baseline** | | | **Post-training** | | |  |
| **Variables** | **N** | **Mean** | **SD** | **N** | **Mean** | **SD** | **N** | **Mean** | **SD** | **N** | **Mean** | **SD** |  |
| Leiter-Revised Spatial Memory | 32 | 17.84 | 9.05 | 32 | 18.84 | 6.25 | 32 | 13.91 | 10.69 | 31 | 19.39 | 11.94 |  |
| Stanford Binet-5 Block Span | 32 | 8.50 | 4.01 | 32 | 9.41 | 4.12 | 32 | 7.19 | 3.83 | 32 | 8.91 | 4.45 |  |
| **Visual Working Memory Composite (Mean)** | **32** | **13.17** | **5.92** | **32** | **14.13** | **4.26** | **32** | **10.55** | **6.65** | **32** | **14.26** | **7.42** | **0.029** |
| Digit Span | 31 | 6.61 | 3.42 | 31 | 6.77 | 3.04 | 31 | 5.00 | 3.37 | 31 | 5.87 | 3.68 | 0.285 |
| BRIEF Parent -Working Memory | 30 | 22.20 | 3.97 | 31 | 21.61 | 3.84 | 31 | 22.52 | 3.48 | 31 | 22.23 | 3.79 | 0.338 |
| BRIEF Teacher - Working Memory | 18 | 22.78 | 3.86 | 15 | 22.60 | 4.73 | 18 | 24.61 | 5.00 | 17 | 23.65 | 5.67 | 0.493 |
| BRIEF Parent - Global Executive Composite | 28 | 157.46 | 22.59 | 30 | 152.10 | 24.06 | 27 | 157.89 | 26.34 | 30 | 153.87 | 22.81 | 0.368 |
| BRIEF Teacher - Global Executive Composite | 17 | 153.06 | 20.94 | 14 | 157.07 | 25.76 | 17 | 153.18 | 31.68 | 17 | 147.47 | 32.49 | 0.216 |
| Connors Parent - Inattention | 29 | 19.28 | 5.92 | 29 | 18.03 | 6.68 | 31 | 19.61 | 6.33 | 31 | 18.90 | 6.70 | 0.488 |
| Connors Teacher - Inattention | 18 | 20.94 | 6.68 | 16 | 19.38 | 5.50 | 18 | 20.28 | 7.63 | 16 | 19.06 | 8.31 | 0.501 |
| Connors Parent - Hyperactivity/Impulsivity | 29 | 23.72 | 8.94 | 29 | 21.62 | 10.96 | 31 | 22.16 | 10.25 | 31 | 21.81 | 11.36 | 0.400 |
| Connors Teacher - Hyperactivity/Impulsivity | 18 | 31.06 | 11.37 | 16 | 31.69 | 11.23 | 18 | 28.94 | 15.16 | 16 | 28.88 | 15.06 | 0.927 |
| KiTAP - Distractibility Errors | 25 | 20.32 | 10.90 | 24 | 17.92 | 12.89 | 22 | 18.41 | 11.51 | 23 | 15.00 | 12.75 | 0.713 |
| KiTAP - Alertness SD of RT | 30 | 325.20 | 305.76 | 29 | 275.48 | 204.13 | 27 | 445.26 | 309.84 | 29 | 441.13 | 292.58 | 0.053 |
| KiTAP - Flexibility False Alarms | 24 | 9.79 | 3.82 | 22 | 8.91 | 2.72 | 19 | 11.79 | 1.75 | 20 | 9.85 | 3.70 | 0.347 |
| KiTAP - Go NoGo False Alarms | 29 | 5.83 | 5.51 | 28 | 6.29 | 6.04 | 25 | 4.60 | 6.03 | 28 | 6.86 | 5.71 | 0.227 |
|  | **Non-Adaptive** | | | | | | **Adaptive** | | | | | | **P-value** |
| **(B) Cogmed version RM** | **Baseline** | | | **Post-training** | | | **Baseline** | | | **Post-training** | | |  |
| **Variables** | **N** | **Mean** | **SD** | **N** | **Mean** | **SD** | **N** | **Mean** | **SD** | **N** | **Mean** | **SD** |  |
| Leiter-Revised Spatial Memory | 18 | 27.83 | 8.47 | 17 | 30.65 | 10.95 | 18 | 32.06 | 11.86 | 18 | 32.67 | 12.06 |  |
| Stanford Binet-5 Block Span | 18 | 14.50 | 2.92 | 17 | 15.88 | 2.98 | 18 | 15.56 | 3.13 | 18 | 17.28 | 3.43 |  |
| **Visual Working Memory Composite (Mean)** | **18** | **21.17** | **4.37** | **17** | **23.26** | **6.49** | **18** | **23.81** | **6.45** | **18** | **24.97** | **7.31** | **0.194** |
| Digit Span | 18 | 10.44 | 3.90 | 17 | 11.53 | 3.20 | 18 | 11.83 | 3.29 | 18 | 13.11 | 3.88 | 0.849 |
| BRIEF Parent -Working Memory | 18 | 20.28 | 3.72 | 16 | 18.44 | 4.26 | 18 | 21.44 | 4.42 | 18 | 20.22 | 4.58 | 0.140 |
| BRIEF Teacher - Working Memory | 10 | 19.40 | 7.72 | 8 | 17.50 | 5.83 | 13 | 21.08 | 5.09 | 13 | 19.15 | 5.21 | 0.260 |
| BRIEF Parent - Global Executive Composite | 18 | 142.50 | 17.46 | 16 | 130.00 | 19.76 | 18 | 141.61 | 27.31 | 17 | 138.00 | 28.35 | 0.011 |
| BRIEF Teacher - Global Executive Composite | 10 | 126.70 | 41.29 | 8 | 116.63 | 32.45 | 12 | 130.50 | 29.38 | 13 | 125.23 | 32.27 | 0.774 |
| Connors Parent - Inattention | 18 | 15.89 | 5.32 | 17 | 14.35 | 6.29 | 18 | 17.39 | 6.21 | 18 | 15.17 | 6.92 | 0.847 |
| Connors Teacher - Inattention | 10 | 13.60 | 11.24 | 9 | 10.56 | 8.99 | 13 | 15.00 | 8.51 | 14 | 12.07 | 7.36 | 0.672 |
| Connors Parent - Hyperactivity/Impulsivity | 18 | 9.39 | 7.39 | 17 | 8.94 | 9.41 | 18 | 12.33 | 11.71 | 18 | 12.67 | 12.66 | 0.340 |
| Connors Teacher - Hyperactivity/Impulsivity | 10 | 15.50 | 15.39 | 9 | 13.67 | 11.21 | 13 | 14.54 | 14.83 | 14 | 12.36 | 12.85 | 0.860 |
| KiTAP - Distractibility Errors | 18 | 12.00 | 9.46 | 16 | 10.19 | 7.48 | 18 | 11.61 | 7.17 | 18 | 11.11 | 9.25 | 0.443 |
| KiTAP - Alertness SD of RT | 18 | 98.47 | 59.65 | 16 | 148.69 | 189.09 | 18 | 194.03 | 193.20 | 18 | 98.14 | 67.55 | 0.251 |
| KiTAP - Flexibility False Alarms | 18 | 5.72 | 3.77 | 16 | 4.69 | 3.79 | 18 | 6.56 | 3.68 | 18 | 4.94 | 3.92 | 0.462 |
| KiTAP - Go NoGo False Alarms | 18 | 1.94 | 1.86 | 16 | 2.56 | 2.83 | 16 | 3.06 | 2.44 | 18 | 3.00 | 2.61 | 0.697 |
| * Results adjusted for active training time |  |  |  |  |  |  |  |  |  |  |  |  |  |
|  |  |  |  |  |  |  |  |  |  |  |  |  |  |
