# Supplementary material for: Cognitive training for children and adolescents with fragile X syndrome: a randomized controlled trial of Cogmed
Source: J Neurodev Disord. 2019 Apr 15;11:4. doi: 10.1186/s11689-019-9264-2 (PMC6463634; doi:10.1186/s11689-019-9264-2)
Supplement: Supplementary file 7 — Table S6. Test-retest reliability (intraclass correlation) of outcome measures used in the study (during 3-month follow-up period without training). (DOCX 14 kb) [file 11689_2019_9264_MOESM7_ESM.docx]

Table S6. Test-retest reliability (intraclass correlation) of outcome measures used in the study (during 3-month follow-up period without training).

| **Outcome Measure** | **N** | **ICC** | **95% CI** | **Level of reliability** |
| --- | --- | --- | --- | --- |
| Stanford Binet Block Span | 88 | .887 | .833 - .925 | Good |
| Leiter-R Spatial Memory | 88 | .759 | .640 - .829 | Good |
| Digit Span | 85 | .925 | .886 - .951 | Excellent |
| KiTAP |  |  |  |  |
| Distractibility (Errors) | 56 | .806 | .690 - .882 | Good |
| Flexibility (False Alarms) | 54 | .766 | .628 - .858 | Good |
| Go-NoGo (False Alarms) | 62 | .771 | .647 - .855 | Good |
| Alertness (SD of RT) | 65 | .602 | .423 - .737 | Moderate |
| Conners Rating Scale |  |  |  |  |
| Parent Inattention | 84 | .815 | .729 - .876 | Good |
| Teacher Inattention | 23 | .865 | .709 - .940 | Good |
| Parent Hyperactivity Impulsivity | 84 | .915 | .872 - .944 | Excellent |
| Teacher Hyperactivity Impulsivity | 24 | .939 | .866 - .973 | Excellent |
| BRIEF |  |  |  |  |
| Parent Working Memory | 87 | .805 | .716 - .868 | Good |
| Teacher Working Memory | 25 | .891 | .757 - .951 | Good |
| Parent GEC | 83 | .906 | .858 - .938 | Excellent |
| Teacher GEC | 25 | .859 | .708 - .935 | Good |
